# Enamel remineralization and surface roughness after treatment with herbal-containing toothpastes

**DOI:** 10.4317/jced.58025

**Published:** 2021-09-01

**Authors:** Letícia-Vendrametto Forcin, Thales-de Sá Oliveira, Pedro-Luiz-Santos Tomaz, Marcelo-Henrick-Maia Matochek, Mackeler-Ramos Polassi, Fabiano-Vieira Vilhena, Nádia-da Rocha Svizero, Paulo-Henrique-Perlatti D’Alpino

**Affiliations:** 1 Hospital for Rehabilitation of Craniofacial Anomalies (HRAC-USP), Universidade de São Paulo, Bauru, SP, Brazil; 2 Trials, Research and Product Development, Bauru, S.P., Brazil

## Abstract

**Background:**

Oral care products containing bioactive agents obtained from extracts of plant drugs were launched. This *in vitro* study investigated the effects of herbal-containing toothpastes associated or not with fluoride to remineralize the enamel after cariogenic challenge with pH cycling. The chemical and physical factors of toothpastes and the enamel surface roughness after brushing were also analyzed.

**Material and Methods:**

Sixty bovine enamel blocks were obtained and divided into 3 thirds: intact (untreated), demineralized (artificial caries lesion), and treated (caries lesion, pH cycling, and brushing with toothpastes). Toothpastes containing herbal compounds contained no fluoride [Galla chinensis (GCH)], low-F concentration [D’Or (DOR); Herbal Bliss (HBL)], or a different fluoride type [Elmex Anticaries (EAC)]. The results were compared to NaF-containing toothpastes: 1450 and 5000 ppm. Enamel blocks were brushed with the toothpastes using a pH-cycling model (7 days). The Knoop hardness (25g/10s) of the surface and the longitudinal sections were then evaluated. The percentage of surface hardness recovery (%SHR) was calculated. The enamel surface roughness, pH, particle size, zeta potential, and polydispersity index of toothpaste slurries were also evaluated. Data were statistically analyzed (α=5%).

**Results:**

No significance was observed when %SHR was compared (*p*>0.05). DOR, GCH, and HBL were more effective in remineralizing the enamel subsurface. Significantly higher surface roughness was observed when treated with EAC and GCH (*p*<0.05).

**Conclusions:**

All toothpastes were able to remineralize the enamel, especially the subsurface, with results equal or better than that of standard toothpastes.

** Key words:**Enamel, hardness, roughness, toothpaste, tooth remineralization.

## Introduction

Systematic reviews focusing on caries prevention in both permanent and deciduous teeth demonstrated that a toothpaste should contain as a minimum of 1,000 μg F/g in order to provide a significant anticaries effect (1). For this reason, biomimetic remineralization, an alternative restorative, a methodology that imitates the natural process of mineralization have been developed to boost enamel remineralization (2). Thus, the mineral gain is facilitated by the addition of different boosters or supplements, even when associated with fluoride in low-F applications (3). In this way, several oral-care products have been launched with varied content of different active ingredients and claims promising protection for teeth against enamel demineralization (4). The research on the development of new oral care products aims not only to reduce enamel demineralization and enhance the remineralizing actions of oral care formulations, but also minimizing the adverse side effects of conventional fluoride therapies (5). Despite these improvements, the action of fluoride in remineralization is still considered the gold standard when compared to other remineralization systems (6).

Fluoride toothpastes are claimed to prevent tooth decay by reducing the solubility of the enamel and promoting the remineralization of incipient lesions in comparison to non-fluoride toothpaste (7). In addition, systematic brushing with a fluoride toothpaste is the main non-professional intervention to prevent caries, but the caries-preventive effect varies as a function of different fluoride concentrations in the composition of the products: higher fluoride concentrations were found to positively correlate with increased remineralization effect and caries control (8).

Recently, oral care products containing bioactive agents obtained from extracts of plant drugs were launched (9). Most of herbal products claim to present actions on gingivitis, anti-plaque prevention, or for reducing dentin hypersensitivity (10). Conversely, a few studies have focused on the remineralization effects of toothpastes containing herbal additives in the composition. This *in vitro* study aimed to investigate the remineralization potential of various commercial toothpastes that contain extracts from vegetal drugs and active remineralization components different from that of standard NaF-containing toothpastes. The present study also evaluated the chemical and physical factors of the toothpaste slurries, and the consequences to the enamel surface roughness after brushing associated with the cariogenic challenge of pH cycling. The research hypotheses were: I- the toothpastes containing extracts from vegetal drugs and other active remineralization components will produce a higher remineralization potential of the enamel surface relative to the standard NaF-containing toothpastes; II- the enamel subsurface will be more effectively remineralized when treated with the herbal-containing toothpastes compared to conventional fluoride containing toothpastes; III- the enamel roughness will be greater after treatment with the toothpastes containing extracts from vegetal drugs and other active remineralization components.

## Material and Methods

- Toothpaste selection

 The characteristics of the products evaluated are listed in Table 1. In the present study, a non-fluoride toothpaste (GCH) or toothpastes containing a declared fluoride compound (EAC, amine fluoride) and/or concentration (HBL and DOR, 980 ppm) different from that of standard NaF-containing fluoride toothpastes were selected. All of these toothpastes contain declared additives from extracts of plant drugs. The results were compared to that of 1450-ppm and 5000-ppm NaF toothpastes (control groups).

**Table 1:**
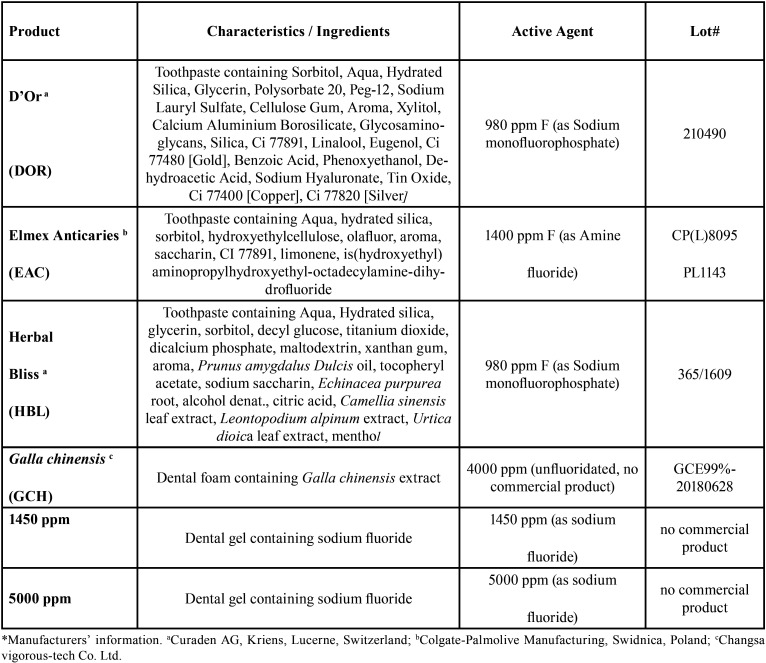
Composition of the toothpastes selected for the study*.

- Chemical and physical analyses of the toothpaste slurries

The pH in toothpaste slurries (1:3, w/v in distilled water) was determined in four different samples of each toothpaste immediately after preparation, using a pH electrode (AK95, Akso, São Leopoldo, RS, Brazil) calibrated with pH 7.0 and 4.0 standards. The particles size distribution, the effective diameter (in nm), and the polydispersity index were also measured at 22°C (Phase Analysis Light Scattering – PALS, 90 Plus, Brookhaven Instruments Corporation, Holtsville, NY, USA) after dilution (final concentration = 250 mg/L) of samples in distilled water (11). Ten replications for the particle analyses were evaluated (n = 10).

- Specimen preparation for the hardness analysis

Sixty enamel blocks (4.0×4.0×6.0 mm) were obtained from bovine incisors using a slow-speed diamond saw (Extec, Enfield, CT, USA) under water-cooling. Enamel blocks were embedded in epoxy resin (EpoxiCure Epoxy Resin and Hardener, Buehler, Lake Bluff, IL, USA). Surfaces were then wet-polished with 600-grit SiC paper (Extec Corp., Enfield, CT, USA) at low speed and with 1,200-grit SiC paper at high speed using a polishing machine (Single Grinder Polisher, Buehler, Lake Bluff, IL, USA). The final polishing was performed with 1-μm diamond paste and wet felt wheels (Extec, Enfield, CT, USA). The sample size for each group was based on a previous study (5) and considering the primary outcomes from surface and cross-sectional hardness analysis considering an estimated surface microhardness (SH) at baseline to select specimens with 285 KHN, a mean standard deviation of 30 KHN, an alpha error of 5% and a beta error of 20% for further experiments. The measurements were performed using a microhardness tester (HMV, Microhardness tester, Shimadzu, Kyoto, Japan) with a Knoop indenter set with 25 g static load for 10 s.

- Caries-Like Lesion Formation

One-third of each surface of the specimens was covered with two consecutive layers of acid-resistant varnish (nail polish, Revlon International Corp., New York, NY, USA), and enamel specimens were subjected to demineralization. To demineralize the enamel and produce caries-like lesions, the specimens were immersed in 30 mL of 50 mM acetate buffer solution containing 3 mM CaCl2.H2O, 3 mM KH2PO4, 50 mM lactic buffer, and 6 μM methylhydroxydiphosphate (MHDP), at pH 5.0 for 5 days. Then, the enamel surface was covered again to leave only 1/3 of its area exposed, and specimens were treated with different toothpaste slurries. In this way, each enamel block surface was divided into three thirds: 1) intact mineralized third was covered with acid-resistant varnish and not exposed to the demineralization solution nor toothpaste treatments; 2) demineralized third–after demineralization, this area was protected with an acid-resistant varnish, as an impeditive for the contact with the toothpastes; and 3) remineralized third – demineralized and treated with toothpaste slurries.

- pH cycling

After demineralization and covering 2/3 of the enamel surface with an acid-resistant varnish, specimens were randomly distributed according to the treatment (n = 8). All of the specimens were submitted to pH cycling alternating demineralization (1.5 mM CaCl2, 0.9 mM KH2PO4, 50 mM lactic buffer, pH 5.0, 8 h) and remineralization solutions (5 mM CaCl2, 0.9 mM KH2PO4, 130 mM KCl, 20 mM HEPES, 5 mM NaN3, pH 7.0, 16 h) for seven days. During the pH cycling, prior to incubation with the remineralization solution, enamel surfaces were treated with 30 mL of each one of the toothpaste slurries in a brushing machine. The enamel blocks were brushed with toothpaste/deionized water slurries (1:3 w/w, 2 mL/block) in a Brushing Simulator Machine MEV-2T (Odeme, Joaçaba, SC, Brazil) at a mean temperature of 37 °C and 120 cycles per minute. A conventional toothbrush with soft bristles (Colgate Classic, Colgate, São Paulo, SP, Brazil) was adapted to the simulator, which brushed the specimens in linear movements with an axial load of 200 g for 5 min. Between the steps, specimens were water rinsed with deionized water.

- Dentin hardness analysis

After pH cycling, enamel surface hardness (SH2) was determined using the same parameters above. For this determination, the acid-resistant varnish was removed, and each of the three thirds of each specimen was tested, applying the indenter in the center of the area, with a distance of 100 μm between each indentation. Ten indentations were carried out in each area (n = 10). The percentage of surface hardness recovery was calculated as follows (5): %SHR = ((SH2 – SH1) / (SH – SH1)) x100) (1)

- Cross-sectional hardness (CSH)

After SH analysis, specimens were longitudinally sectioned using a water-cooled rotating diamond wheel at a low speed, and both half-blocks were used for CSH measurements. For that, enamel halves were embedded in epoxy resin and polished, as previously described. Three series of indentations were performed at eight different depths from the enamel surface (10, 30, 50, 70, 90, 110, 220, and 330 μm) in the central region of each third. The indentations were spaced 100 μm from each other.

- Surface roughness (SR)

Specimens were individually positioned in a surface roughness tester (Surftest 401; Mitutoyo, Kawasaki, Japan) to measure the roughness average (Ra) values. Three readings were made in the treated, remineralized thirds of the sample (n=3), and the mean surface roughness (Ra) was determined and the results compared. The extension of each reading was 2.85 mm, using a cutoff of 0.8 mm.

- Surface morphology

Two additional specimens of each group were prepared for SEM (JSM-5310; JEOL, Tokyo, Japan) analysis. Specimens were sputter-coated with gold in a vacuum evaporator (MED 010; Balzers, Balzer, Liechtenstein), and photomicrographs of surface micromorphology of demineralized and remineralized thirds were obtained at 5,000× magnification.

- Statistical analysis

Data were calculated and statistically analyzed with a statistics software (Statsoft, Tulsa, OK, USA). Data were subjected to one-way analysis of variance, followed by Tukey post hoc test, at a presel alpha of 0.05.

## Results

The results of the chemical and physical properties of the toothpaste slurries are depicted in Table 2. Most of the toothpastes presented nearly neutral pH. Only the toothpastes EAC and GCH were acidulated (4.76 and 4.65, respectively). The particle size varied from 321.1 nm (HBL) to 562.2 nm (GCH). GCH presented significantly larger particle size in comparison to all other products. The zeta potential varied from -0.62 mV (GCH) to 0.19 mV (HBL). Despite the difference among the potential zeta means, no significance was observed when comparing the means. The polydispersity index varied from 0.17 (HBL) to 0.34 (DOR and GCH). The polydispersity indexes observed for DOR, GCH, 1450 ppm, and 5000 ppm were significantly higher than that of observed for EAC and HBL.

**Table 2:**
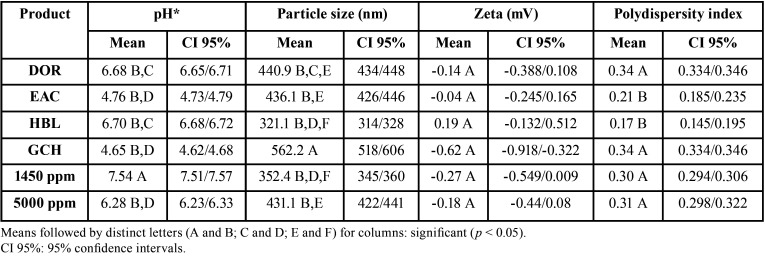
Results of the chemical and physical analyses of the toothpaste slurries.

The results of the SH analyses are presented in Table 3. In the mineralized dentin, the SH means varied from 243.2 (DOR) to 306.1 KHN (GCH), which presented significantly higher SH means (*p*>0.05). No significance was also found when the SH1 means were compared, varying from 187.8 (DOR) TO 241.5 KHN (GCH). The highest surface hardness after pH cycling associated with the toothpastes tested (SH2) occurred when treated with GCH (336.8 KHN) and the lowest average when treated with DOR (256.7 KHN). GCH also favored the highest %SHR, with a surface recovery of 147.2%. Conversely, the lowest recovery in surface hardness was observed when treated with EAC toothpaste (119.5%). The statistical analysis demonstrated no significant differences when means of %SHR were compared to the treatments with the control groups (*P* <0.05). All of the toothpastes tested remineralized the demineralized third more than 100%, which was demonstrated in the calculations of the percentage of surface hardness recovery (Table 3).

**Table 3:**
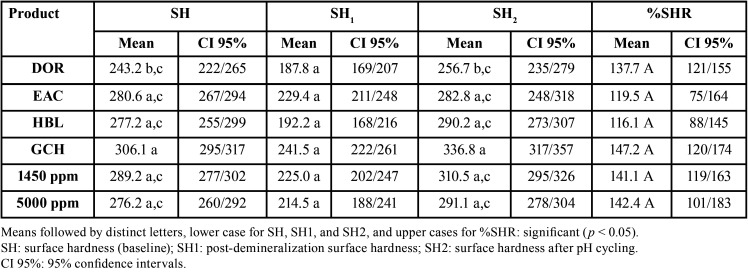
Results of surface hardness analysis (n=10) according to the different treatments.

**Figure 1:**
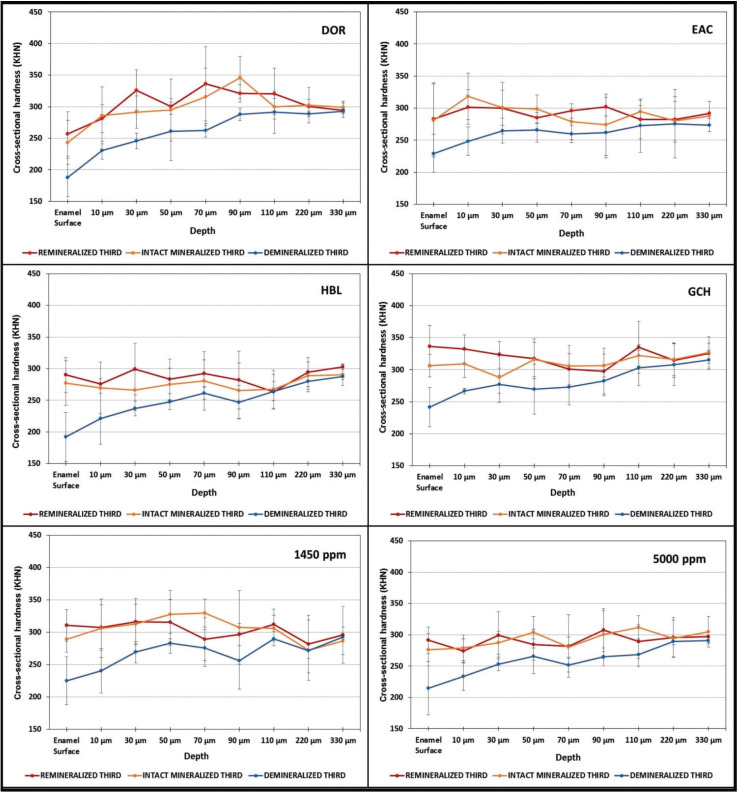
Cross-sectional hardness (means) at different depths in enamel blocks as a function of the enamel third (intact, demineralized, and remineralized). The bars denote standard deviations.

Figure 1 displays the variations in the cross-sectional hardness according to the depth and the evaluated enamel areas (intact, demineralized, and remineralized thirds). Lower hardness means was clearly observed in the demineralized thirds in all conditions in comparison to that of the intact, untreated third, exhibiting a wider drop in the shallower subsurface lesion depths. For most of the toothpastes, an overlap of the means was clearly observed when comparing the cross-sectional hardness means of the intact and treated conditions (Fig. 1). This was particularly true for the toothpastes EAC, 1450 ppm, and 5000 ppm. In addition of the superficial hardness recovery of more than 100%, the cross-sectional hardness was also positively impacted, demonstrating that the potential of these toothpastes to aid the remineralization of the subsurface lesion in relation to the demineralized condition. The most effective recovery of the cross-sectional hardness was found for DOR, HBL, and GHC, which were able to reverse the lesion formed to a depth of 80, 110, and 50 μm, respectively (Fig. 1).

The results of enamel surface roughness after brushing associated with the toothpastes are displayed in Table 4. The lowest enamel surface roughness was observed after brushing with HBL (0.193), and the highest with GCH (0.332). Brushing with the toothpaste during the pH cycling significantly affected the enamel roughness when treated with EAC and GCH, which exhibited the highest Ra means (*p*<0.05).

**Table 4:**
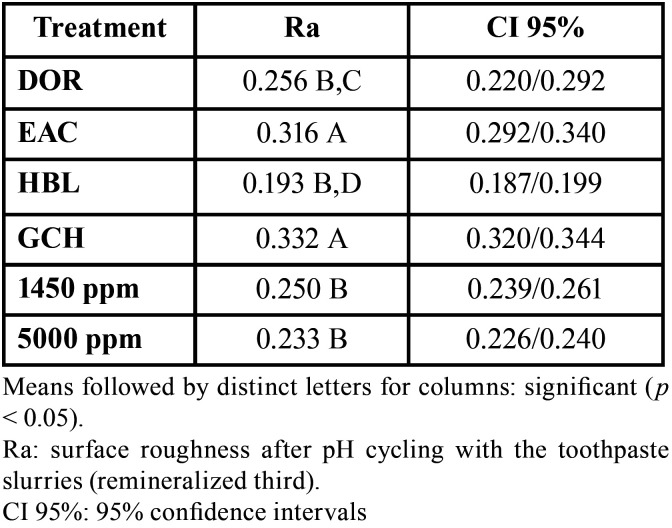
Results of surface roughness (Ra) of the remineralized third according to the treatments (n=3).

**Figure 2:**
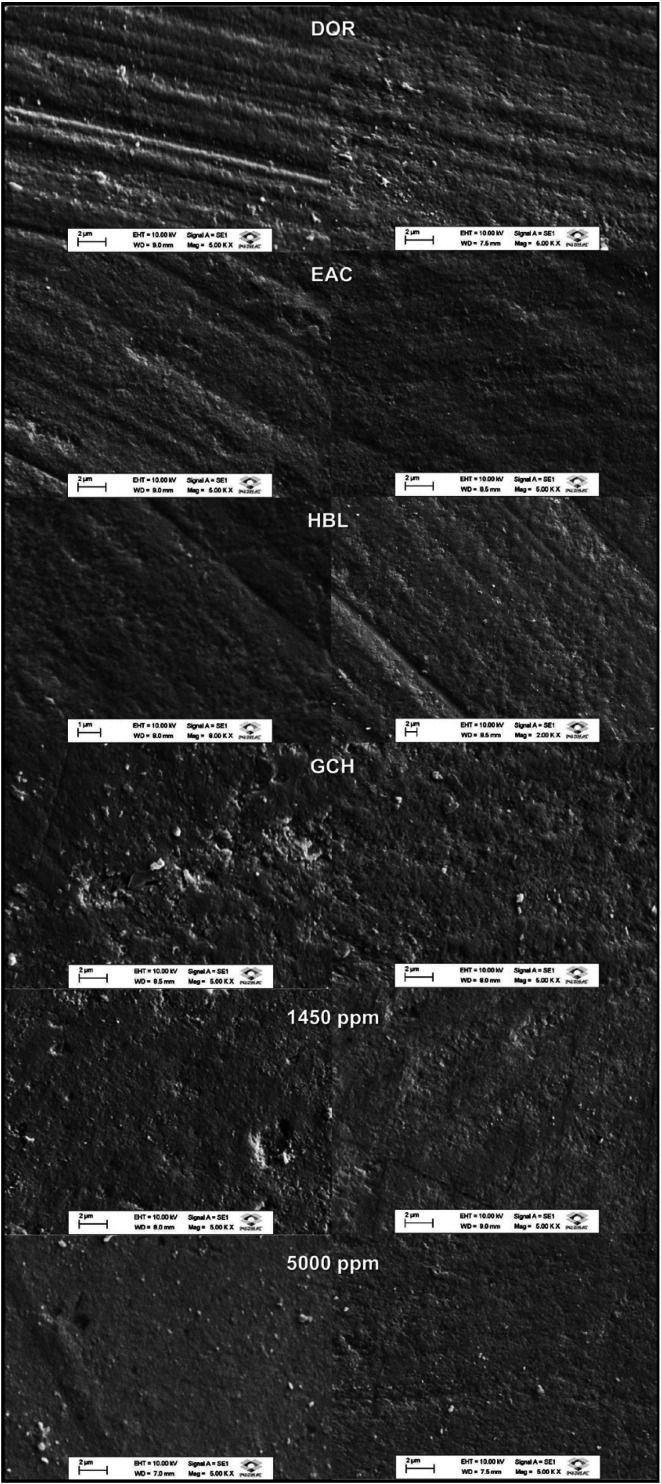
Representative photomicrographs (SEM analysis) of the demineralized (left) and remineralized, treated thirds (right). 5.000x magnification.

Figure 2 displays the surface morphology of the treated, remineralized thirds according to the experimental groups. There was a change in the surface morphology after brushing clearly demonstrated when comparing the images of the remineralized thirds (right side) with the demineralized one (left side). Despite the differences in the initial morphology among the experimental groups, most of the remineralized areas exhibited smother surface than the demineralized third.

## Discussion

The results of this *in vitro* study showed that the toothpastes favored the mineral gain at the enamel surface when treating the demineralized enamel surface. All the experimental groups favored a recovery of enamel surface hardness above 100%. In this manner, the first hypothesis which anticipated that the toothpastes containing extracts from vegetal drugs and other active remineralization components would produce a higher remineralization potential of the enamel surface relative to the standard NaF-containing toothpastes, was accepted. Although all toothpastes were effective in remineralizing the enamel surface, DOR, HBL, and GCH were more effective in remineralizing the enamel subsurface. These toothpastes promote the deepest subsurface remineralization effect (to a depth of 110 µm), in comparison to the intact third. When toothpaste GCH was used, the mean %SHR was the highest (147.2%) and the cross-sectional hardness means of the remineralized third were higher than that of the intact third up to approximately 50 µm deep. To a lesser magnitude at the enamel surface, DOR and HBL also proved to effective products for remineralizing both the subsurface enamel. Conversely, the means of the cross-sectional hardness of the intact and remineralized thirds when treated with toothpastes 1450 and 5000 ppm overlapped throughout the deeper areas (Fig. 1). For the toothpaste EAC, which was as effective in remineralizing the enamel surface when compared to other products, the means of the cross-sectional hardness were equal or lower than the means of the intact third up to 60 µm (Fig. 1). Despite these differences, this *in vitro* study showed that the all toothpastes had a significant effect on reversing the caries-like lesion at the enamel surface. In addition, all toothpastes also favored the subsurface mineral gain when treating the demineralized enamel as the cross-sectional hardness of the remineralized third was higher than that observed in the demineralized third throughout the analyzed depths. In this way, the second hypothesis, which anticipated that the herbal-containing toothpastes would be more effective to remineralize the enamel subsurface when compared to conventional fluoride containing toothpastes, was not accepted.

It is important to highlight that the toothpastes selected for this *in vitro* study contain no fluoride (GCH) or contain a declared a fluoride compound (amine fluoride) and/or concentration (980 ppm) different from that of standard 1450-ppm fluoride-containing toothpastes. EAC contains limonene, the major lemon oil constituent of *Citrus limon L.*, found to present antimicrobial activity against several oral pathogens, especially against *Streptococcus mutans* (12). In addition, amine fluoride is the bioactive agent in a concentration of 1400 ppm, according to the manufacturer. In a previous *in vitro* study (13), the remineralizing effect of enamel toothpastes with differing fluoride compounds found that amine fluoride presented higher remineralizing effect when compared to formulations containing sodium fluoride and sodium monofluorophosphate.

EAC also contains olafluor, an acidic agent consisting of a salt of an alkyl ammonium cation and fluoride. It is regarded to form a film layer on the enamel surface, which facilitates the incorporation of fluoride into the enamel tissue, forming a less soluble fluorapatite at the subsurface. Concerns have been expressed over the remineralization effect of olafluor at the enamel subsurface as fluoridation reaches only a depth of a few nanometers, only producing “ultrathin fluorapatite layers” (14). This problem was highlighted, as the mechanism of the formation of fluorapatite proposed seems to be inaccurate when olafluor is present in the formulation. In the present study, although EAC was able to recover the surface hardness in more than 100%, the cross-sectional hardness demonstrated overlapping hardness means, with similar or lower means at the enamel subsurface up to 50 µm in comparison with the intact third (Fig. 1).

The toothpaste DOR contains a declared content of sodium fluoride associated with, among other compounds, a proprietary technology containing eugenol and linalool, essential oils derived from the medicinal plant *Thymus vulgaris* L. type (15). Both phyto-compounds present phenolic groups with antimicrobial activity (15). It also contains sodium hyaluronate, the sodium salt of hyaluronic acid, a glycosaminoglycan found in various connective tissues of humans. Hyaluronic acid-based gel formulations were found to present antibacterial effects against oral bacteria (16). Based on the manufacturer’s technical profile, there is no clear information as to which specific compound would be the remineralizing agent in the toothpaste DOR. It can be speculated that this technology seems to be based on the presence of glycosaminoglycans, which tends to aggregation around a protein core to form a proteoglycan molecule, a noncollagenous protein. Proteoglycans and other SIBLING non-collagenous proteins were regarded to control several aspects of the mineralization process in mineralized tissues (tooth and bone tissues) (17). Depending on the protein concentration and availability to react, non-collagenous proteins may affect the mechanism of calcium precipitation in biomimetic remineralization models (18). It has been also pointed out that noncollagenous peptides, in self-organizing assemblies, can also induce hydroxyapatite growth, decreasing the enamel demineralization (19). Despite the manufacturer’s commercial appeal for containing 23.75 carat gold dust, the addition of glycosaminoglycans associated with sodium monofluorophosphate seems to be responsible minerals to boost the remineralization process. This association possibly allowed adequate levels of calcium and phosphate ions in association with the fluoride ions, which positively affected the remineralization of hydroxyapatite crystals of the enamel surface and within the subsurface carious lesion.

The toothpaste containing herbal ingredients was as effective in remineralizing the enamel surface and subsurface. HBL is produced and marketed by the same manufacture as DOR toothpaste. Its proprietary formulation contains, according to the technical profile, *Prunus amygdalus Dulcis* oil, *Echinacea purpurea* root, *Camellia sinensis* leaf extract, *Leontopodium alpinum* extract, and *Urtica dioica* leaf extract, among other compounds. *E. purpurea* and *L. alpinum*, popularly known as Edelweiss, are medicinal herbals with antimicrobial actions against different pathogens (20). Conversely, U. dioica was found to present wound healing properties due to the presence of flavonoids and polyphenols.(21) The remineralizing effectiveness of HBL seems to be related to synergic actions of fluoride content with the presence of *Prunus amygdalus Dulcis* and *Camellia sinensis*. *P. amygdalus Dulcis*, popularly known as sweet almond, which contains varied concentrations of proanthocyanidins and hydrolysable tannins (such as ellagic and gallic acids) (22). It also contains ions varied concentrations of calcium, magnesium, phosphorous, and vitamin E (23). *C. sinensis* is the botanical name of green tea, known to present beneficial effects on oral health. The main components of *C. sinensis* are polyphenols with the main types as being catechins (24). Minerals and trace elements such as calcium, phosphorus, fluoride, among others, were also described in the composition. It is also known for its antimicrobial action against *S. mutans*, preventing enamel demineralization.

**Figure 3:**
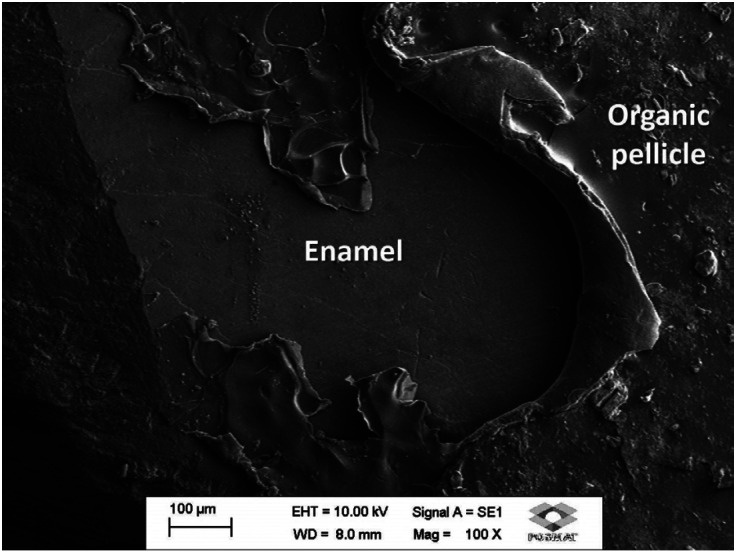
Representative photomicrophaph of the organic pellicle formed after application of Galla chinensis-containing dentifrice. 100x magnification.

*Galla chinensis* dentifrice tested in the present study contained no fluoride in the composition. *Galla chinensis* is a rich natural product containing polyphenols (e.g. gallotannins and gallic acid) and other components, such as carbohydrates, proteins, and other constituents (25). The results of the present study also demonstrated that dentifrice containing *Galla chinensis* was the most effective product to recover the hardness at the enamel surface. Although no significance was observed when the experimental groups were compared, *Galla chinensis* presented the highest percentage of enamel surface recover (147.2%). At the subsurface, it was as effective to remineralize the subsurface promoting mineral gain, which was demonstrated by the means hardness above 300 KHN up to 70 µm deep. The SEM analysis also demonstrated that *Galla chinensis* forms an organic pellicle on the enamel surface (Fig. 3). This pellicle may be formed by quantities of monomeric and polymeric polyphenols (26). This seems to favor an increased contact of the pellicle-containing gallotannins with the enamel surface, increasing its effectiveness to remineralize the enamel.

The results of the present study were corroborated in a previous study in which *Galla chinensis* proved to be effective to promote enamel remineralization (27). In another study (28), it was found that *Galla chinensis* was able to nucleate mineral ions deposits in the body of lesion, rather than on the surface of lesion when analyzed using transverse microradiography. Despite the effectiveness of *Galla chinensis* in enhancing the remineralization of dental caries, its remineralization mechanism is still unclear and seems to be different from that of fluoride (27).

It is true that fluoride treatments are generally effective in helping to protect the dental enamel from demineralization and enhancing remineralization. Calcium salts or calcium containing materials have been developed to enhance the delivery and retention of fluoride into the oral cavity. Recently, non-fluoride systems were developed in order to boost the remineralization process. Despite this evolving technology, fluoride-containing oral care products are still considered gold standard agents. These results were corroborated by the results of the fluoride-containing 1450 and 5000-ppm experimental groups. Both fluoride toothpastes were effective to remineralize the enamel surface. The cross-sectional hardness results also demonstrated that these two toothpastes were able to recover the hardness with means, which overlapped with that of the hardness means of the intact third in the deeper areas, especially for 5000-ppm fluoride-containing toothpaste.

In addition to these differences, another important factor related to the effectiveness of fluoride toothpastes is related to the pH of the products. The toothpastes EAC and GCH presented the most acidic pH. It is important to highlight that the toothpaste slurries were prepared with water, which maintained the low pH of the acidulated dentifrice slurries. The surface roughness of the remineralized third varied as a function of the treatment with the chemical and physical characteristics of the toothpastes tested. Toothpastes with acidic pHs significantly affected the surface roughness. The zeta potential varied as a function of the toothpastes with none of them displaying a tendency to aggregation as not of them presented electrically neutral zeta potential (29). GCH presented the biggest particle size and EAC was the second one. It seems that the synergism with the pH negatively affected the surface roughness of the treated enamel. Thus, the third hypothesis which anticipated that the enamel roughness would be greater after treatment with the toothpastes containing extracts from vegetal drugs and other active remineralization components was partially accepted. Only the enamel treatments with EAC and GCH significantly increased the enamel roughness.

## Conclusions

From the analysis of the results of the present study, it can be concluded that all toothpastes tested were able to effectively recover the enamel surface hardness. Also, the cross-sectional hardness varied depending on the toothpaste used to treat the remineralized third. For most of the products tested, a relationship between surface remineralization and subsurface remineralization occurred. The chemical and physical parameters associated interfere with the enamel surface roughness, as moderate correlations were found when these parameters were analyzed separately versus enamel roughness.
